# HTX-011 reduced pain intensity and opioid consumption versus bupivacaine HCl in herniorrhaphy: results from the phase 3 EPOCH 2 study

**DOI:** 10.1007/s10029-019-02023-6

**Published:** 2019-08-19

**Authors:** E. Viscusi, H. Minkowitz, P. Winkle, S. Ramamoorthy, J. Hu, N. Singla

**Affiliations:** 1 Sidney Kimmel Medical College of Thomas Jefferson University, 111 South 11th Street, Gibbon Building, Suite 8490, Philadelphia, PA 19107 USA; 2 HD Research Corp, Houston, TX USA; 3 Anaheim Clinical Trials, Anaheim, CA USA; 4 University of California at San Diego Health System, San Diego, CA USA; 5 Heron Therapeutics, Inc., San Diego, CA USA; 6 Lotus Clinical Research, LLC, Pasadena, CA USA

**Keywords:** Inguinal hernia repair, Postoperative pain, Pain management, Multimodal analgesia, HTX-011, Opioid sparing

## Abstract

**Purpose:**

Currently available local anesthetics have not demonstrated sufficient analgesia beyond 12–24 h postoperatively. The purpose of the study was to assess the safety and efficacy of HTX-011 (bupivacaine and meloxicam in Biochronomer^®^ polymer technology), a long-acting investigational anesthetic, in reducing both postoperative pain over 72 h and postoperative opioid use compared to bupivacaine hydrochloride (HCl).

**Methods:**

A phase 3, randomized, double-blind, active-controlled multi-center study (EPOCH 2; NCT03237481) in subjects undergoing unilateral open inguinal herniorrhaphy with mesh placement was performed. Subjects randomly received a single intraoperative dose of HTX-011, immediate-release bupivacaine HCl, or saline placebo prior to closure.

**Results:**

The study evaluated 418 subjects, and the primary and all key secondary efficacy endpoints were in favor of HTX-011. HTX-011 reduced mean pain intensity by 23% versus placebo (primary endpoint; *p* < 0.001) and by 21% versus bupivacaine HCl (*p* < 0.001) with significant reductions in the number of patients experiencing severe pain. Opioid consumption over 72 h was reduced by 38% versus placebo (*p* < 0.001) and 25% versus bupivacaine HCl (*p* = 0.024). Overall, 51% of HTX-011 subjects were opioid-free through 72 h (versus 22% for placebo [*p* < 0.001] and 40% for bupivacaine HCl [*p* = 0.049]). HTX-011 was generally well-tolerated with fewer opioid-related adverse events reported compared to the bupivacaine HCl and placebo and no evidence of local anesthetic systemic toxicity.

**Conclusions:**

HTX-011 demonstrated significant improvement in postoperative pain control and a clinically meaningful reduction in opioid consumption when compared to the most widely used local anesthetic, bupivacaine HCl.

## Introduction

Herniorrhaphy is one of the most commonly performed surgeries with more than 20 million performed annually worldwide [1]. Up to 70% of subjects experience moderate to severe pain after surgery [2–4] with the greatest degree of pain occurring within the first 72 h [5]. Standard clinical practice for managing postoperative pain includes preoperative and intraoperative use of local infiltration and/or anesthetic field blocks with local anesthetics such as bupivacaine [1], which is the most widely used local anesthetic for implementing postoperative analgesia. Unfortunately, bupivacaine has limited efficacy beyond 6–12 h, which frequently results in the overreliance on opioids for postoperative pain management [6–8]. Although opioids can provide effective analgesia for moderate to severe pain, higher opioid doses are associated with an increased risk of adverse drug effects (including postoperative nausea and vomiting, respiratory depression, sedation, and delirium/confusion) [9, 10]. These adverse events (AEs), along with poorly controlled pain, have been shown to contribute directly to patient discomfort, dissatisfaction, delayed recovery from surgery, increased length of hospital stay, and increased medical costs [11–15]. Moreover, opioids are associated with serious risks of misuse, abuse, addiction, and diversion [16]. Alternative long-acting, non-opioid analgesics that provide postoperative analgesia throughout the first 72 h would enhance multimodal regimens that aim to reduce or eliminate the need for opioids.

HTX-011 is a novel, extended release, fixed-dose combination local anesthetic comprising bupivacaine and low-dose meloxicam, incorporated in a proprietary Biochronomer^®^ polymer. After single-dose administration, the polymer enables extended release of bupivacaine and meloxicam simultaneously over approximately 3 days. Low-dose meloxicam has been shown to reduce local inflammation [17], which may help normalize changes in local pH caused by surgery [18]. Based on previous research in bupivacaine [19, 20], inclusion of meloxicam in HTX-011 to produce these effects locally may allow enhanced penetration of bupivacaine into the nerves in the days following surgery, thereby potentiating its analgesic effect to reduce pain more effectively than the summed effects of each component alone. In a prior phase 2 herniorrhaphy study, the same low dose of meloxicam alone in the polymer formulation locally administered into the surgical site produced no direct analgesic effect and demonstrated synergy with bupivacaine to produce significantly greater pain reduction than the polymer formulation containing only bupivacaine [21].

This randomized, double-blind, placebo- and active-controlled phase 3 study (EPOCH 2) was designed to evaluate the analgesic efficacy and safety of HTX-011 (300 mg/9 mg) administered as a single dose into the surgical site compared with bupivacaine hydrochloride (HCl) and saline placebo in subjects undergoing unilateral open inguinal herniorrhaphy with mesh placement.

## Methods

The study (ClinicalTrials.gov, NCT03237481) was conducted at 16 sites across the United States and 1 site in Belgium from July 2017 through January 2018. The study protocol was approved by the institutional review board/international ethics committee for each center and all subjects included in this study provided written informed consent.

Eligible subjects were required to be at least 18 years of age with an American Society of Anesthesiologists physical status of I, II, or III. The study excluded subjects with a pre-existing, concurrent acute, or chronic painful physical/restrictive condition that could confound the postoperative assessments and excluded subjects with planned or concurrent surgical procedures and those with a history of prior inguinal herniorrhaphy except during childhood. Those with known or suspected daily use of opioids for 7 or more consecutive days within 6 months prior to their scheduled surgery were also excluded. Other key exclusion criteria include the use prior to the scheduled surgery of non-steroidal anti-inflammatory drugs (NSAIDs, including meloxicam) within 10 days, long-acting opioids within 3 days, any opioids within 24 h, bupivacaine within 5 days, and systemic steroids within 10 days.

The study employed a double-blind design wherein neither the patient nor the investigators involved in conducting postsurgical assessments were aware of the treatment given. Subjects were randomized to the following three treatment groups in a 2:2:1 ratio: (a) HTX-011, 300 mg/9 mg (bupivacaine/meloxicam), 10.3 mL, via instillation into the surgical site; (b) bupivacaine HCl 0.25%, 75 mg (30 mL), via injection into the surgical site; (c) saline placebo, 10.3 mL, via instillation into the surgical site. Randomization was performed using a centralized computer-generated blocked randomization algorithm created by an interactive response technology (IRT) provider. On the day of surgery (Day 1), subjects underwent a unilateral open inguinal herniorrhaphy with mesh placement under general anesthesia. Spinal, epidural, or regional anesthesia was not permitted. Intraoperative administration of opioids (other than intravenous [IV] fentanyl) or other analgesics was prohibited.

Near the completion of surgery and following irrigation and suction of each fascial layer, a single dose of study drug (HTX-011, bupivacaine HCl, or saline placebo) was administered intraoperatively via local administration into the surgical site. Subjects remained in the hospital/research facility for a minimum of 72 h following surgery. Subjects could only receive specific rescue medication upon request to treat postoperative pain, not for pain prophylaxis during the 72-h postoperative observation period. Permitted postoperative rescue medication consisted of oral oxycodone (no more than 10 mg within a 4-h period as needed), IV morphine (no more than 10 mg within a 2-h period as needed), and/or oral acetaminophen (no more than 1000 mg in a 6-h window). No multimodal postoperative analgesic regimen was prescribed during the 72-h postoperative observation period, and other analgesics such as NSAIDs were not permitted during the 72-h postoperative observation period. After the 72-h assessments were completed, subjects could be discharged and were instructed to return to the study site on Days 10 and 28 to complete follow-up assessments. Upon discharge, subjects were to complete a daily diary to record if they needed opioid medication after discharge and through Day 28.

### Outcome measures

The primary efficacy endpoint was mean area under the curve (AUC) of the numeric rating scale (NRS) of pain intensity scores through 72 h (AUC_0–72_) for HTX-011 compared with saline placebo. Hierarchical-tested, key secondary endpoints included: (1) mean AUC_0-72_ of the NRS pain intensity scores for HTX-011 compared with bupivacaine HCl, (2) mean total postoperative opioid consumption (in morphine equivalents) through 72 h for HTX-011 compared with saline placebo, (3) the proportion of subjects who were opioid-free through 72 h for HTX-011 compared with bupivacaine HCl, and (4) the mean total postoperative opioid consumption (in morphine equivalents) through 72 h for HTX-011 compared with bupivacaine HCl. To account for multiple hypothesis testing on the primary endpoint and on each of the four key secondary endpoints, a strict testing hierarchy was applied to control study-wise alpha level at 0.05. In this method, subsequent endpoints were tested for significance unless the preceding endpoint in the prespecified order did not reach statistical significance. This method helps control for false positives (i.e., Type I error rate) in statistical testing for multiple hypotheses. Other secondary efficacy endpoints included the proportion of subjects who were opioid-free through 72 h, through Day 10 and through Day 28 compared with saline placebo, and the proportion of subjects with severe pain (defined as an NRS pain intensity score ≥ 7) at any time point through 72 h.

Safety endpoints included the incidence of AEs, change from baseline in clinical laboratory results, electrocardiogram (ECG) data and vital signs, and wound-healing assessments (at 72 h, Day 10, and Day 28). Prespecified analysis was also conducted specifically for opioid-related AEs (ORAEs) through the safety follow-up on Day 28, including AEs that coded to any of the following prespecified terms: nausea, vomiting, constipation, pruritus, somnolence, respiratory depression, or urinary retention.

### Statistical analysis

Based on the previously completed phase 2 study of HTX-011 in subjects undergoing unilateral open inguinal herniorrhaphy with mesh placement [21], a sample size analysis indicated that approximately 400 subjects (160, 160, and 80 in the HTX-011, bupivacaine HCl, and saline placebo groups, respectively) would provide at least 90% power to detect a statistically significant difference between the HTX-011 group and each of the control groups for each of the primary and key secondary endpoints.

The primary and first key secondary endpoints were analyzed using analysis of variance (ANOVA) for the intent to treat population. To adjust for bias in pain scoring due to opioid use, pain intensity observations made after opioid rescue medication use were replaced by the highest pain score recorded before the opioid was given. A prespecified sensitivity analysis of the primary endpoint was also performed with no adjustment for opioid usage. The total postoperative opioid consumption through 72 h was analyzed using a Wilcoxon rank sum test. The proportion of patients who were opioid-free through 72 h was analyzed using Fisher’s exact test. All AEs were coded and tabulated by System Organ Class and Preferred Term.

## Results

### Patient characteristics

**Fig. 1:**
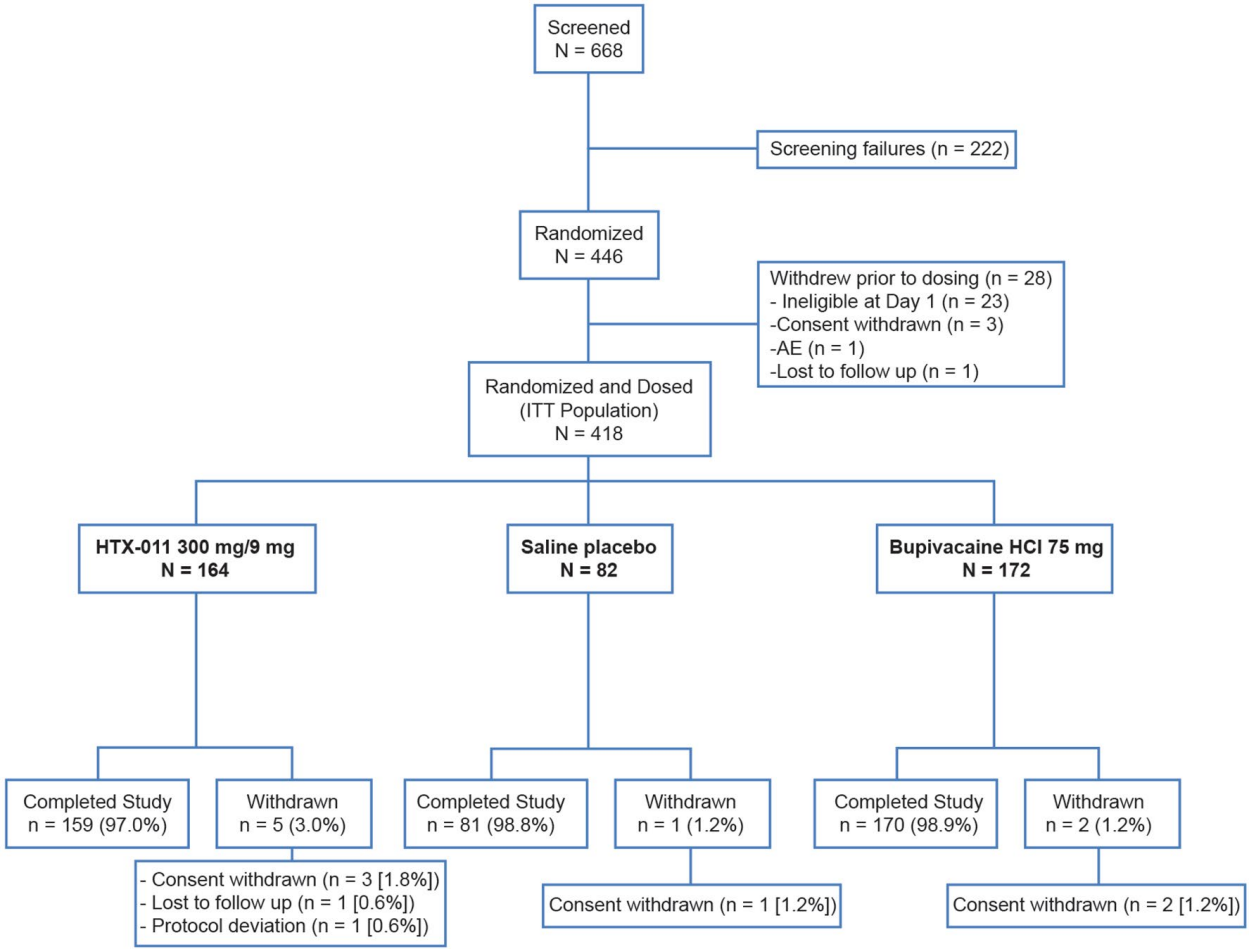
CONSORT flow diagram for EPOCH 2 study. *AE* adverse event, *HCl* hydrochloride, *ITT* intent to treat. Notes: “Screened” was defined as signing an informed consent form. “Completed study” was defined as completing the Day 28 visit. One subject randomized to the HTX-011 group was misdosed; the subject received bupivacaine HCl instead of HTX-011

A total of 446 subjects were randomly assigned to the 3 study groups, of whom 418 received a dose of study drug (ITT population); ineligibility at the time of surgery was the most common reason subjects were not dosed (Fig. 1). Baseline characteristics were similar among the three groups and most subjects were male, as expected given the surgical procedure (Table 1).

**Table 1:** Baseline demographic by study group (ITT population)

Baseline characteristics	Saline placebo (*n* = 82)	Bupivacaine HCl 75 mg (*n* = 172)	HTX-011 300 mg/9 mg (*n* = 164)
Age (years)—mean (SD)	48.0 (14.59)	49.4 (11.26)	48.9 (13.29)
Sex, *n* (%)			
Female	3 (3.7%)	8 (4.7%)	12 (7.3%)
Male	79 (96.3%)	164 (95.3%)	152 (92.7%)
Race, *n* (%)			
American Indian or Alaskan Native	0	0	2 (1.2%)
Asian	1 (1.2%)	2 (1.2%)	2 (1.2%)
Black or African Descent	3 (3.7%)	16 (9.3%)	17 (10.4%)
Native Hawaiian or another Pacific Islander	0	1 (0.6%)	4 (2.4%)
White	78 (95.1%)	153 (89.0%)	139 (84.8%)
Ethnicity, *n* (%)			
Hispanic or latino	30 (36.6%)	51 (29.7%)	43 (26.2%)
Not hispanic or latino	52 (63.4%)	121 (70.3%)	121 (73.8%)
BMI (kg/m^2^)			
Mean (SD)	28.12 (4.232)	26.86 (3.578)	27.14 (4.386)
Median	27.99	26.44	26.90
Min, Max	17.6, 38.5	19.4, 37.9	17.9, 38.5

ITT population includes all subjects who were randomized and received study drug

*BMI* body mass index, *ITT* intent to treat, *SD* standard deviation

### Efficacy

#### Primary and secondary endpoints

The results for the primary and all four key secondary endpoints were statistically significant in favor of HTX-011 (Table 2). HTX-011 demonstrated superior, sustained pain reduction through 72 h, significantly reduced opioid consumption, and resulted in significantly more opioid-free patients compared with bupivacaine HCl and saline placebo (Table 3).

**Table 2:** Efficacy results for the primary and key secondary endpoints (ITT population)

	Saline placebo (*n* = 82)	Bupivacaine HCl 75 mg (*n* = 172)	HTX-011 300 mg/9 mg (*n* = 164)
AUC_0–72_ of the NRS pain intensity scores^a^			
Mean (SD)	350.8 (171.22)	341.9 (158.30)	269.4 (173.72)
Primary endpoint: *p* value^b^ versus saline placebo			0.0004
Secondary endpoint: *p* value^b^ versus bupivacaine HCl			< 0.0001
Opioid consumption through 72 h (MME)			
Mean (SD)	17.5 (18.91)	14.5 (18.19)	10.9 (17.06)
Median (Min, Max)	11.3 (0.0, 73.5)	7.3 (0.0, 87.5)	0.0 (0.0, 103.0)
Secondary endpoint: *p* value^c^ versus saline placebo			0.0001
Secondary endpoint: *p* value^c^ versus bupivacaine HCl			0.0240
Opioid-free through 72 h			
* n* (%)	18 (22.0%)	69 (40.1%)	84 (51.2%)
* p* value^c^ versus saline placebo			< 0.0001
Secondary endpoint: *p* value^d^ versus bupivacaine HCl			0.0486

Opioid-free through 72 h is defined as subjects who had total MME opioid dose = 0 from 0 to 72 h. All doses of opioid rescue medication are expressed as intravenous MME

ITT population includes all subjects who are randomized and receive study drug. This population was used as the primary analysis population for all efficacy endpoints

*AUC*_*0–72*_ area under the curve through 72 h, *HCl* hydrochloride, *ITT* intent to treat, *MME* morphine milligram equivalent, *NRS* Numeric Rating Scale of the pain intensity score, *SD* standard deviation, *wWOCF* windowed worst observation carried forward

^a^Analyzed using wWOCF

^b^*p* values reflect results of an analysis of variance (ANOVA) with randomized treatment as the main effect

^c^*p* values were obtained using the Wilcoxon rank sum test

^d^*p* values from Fisher’s exact test

**Table 3:** Mean AUC of the NRS of pain intensity over time using wWOCF (ITT population)

	Saline placebo (*n* = 82)	Bupivacaine HCl 75 mg (*n* = 172)	HTX-011 300 mg/9 mg (*n* = 164)
AUC_0–8_			
Mean (SD)	50.0 (15.03)	34.4 (16.31)	30.6 (18.87)
* p* value versus saline placebo		< 0.0001	< 0.0001
* p* value versus bupivacaine HCl			0.0426
AUC_0–12_			
Mean (SD)	75.3 (24.59)	57.7 (24.88)	46.3 (29.31)
*p* value versus saline placebo		< 0.0001	< 0.0001
*p* value versus bupivacaine HCl			0.0001
AUC_0–24_			
Mean (SD)	143.8 (54.94)	126.7 (52.68)	97.7 (60.31)
*p* value versus saline placebo		0.0238	< 0.0001
*p* value versus bupivacaine HCl			< 0.0001
AUC_24–72_			
Mean (SD)	207.1 (122.32)	215.2 (111.97)	171.7 (120.40)
*p* value versus saline placebo		0.6041	0.0264
*p* value versus bupivacaine HCl			0.0007
AUC_0–72_			
Mean (SD)	350.8 (171.22)	341.9 (158.30)	269.4 (173.72)
*p* value versus saline placebo		0.6902	0.0004
*p* value versus bupivacaine HCl			< 0.0001

ITT population includes all subjects who are randomized and received study drug. Analyzed using windowed worst observation carried forward (wWOCF). *p* values reflect results of an analysis of variance (ANOVA) with randomized treatment as the main effect

*AUC* area under the curve, *HCl* hydrochloride, *ITT* intent to treat, *NRS* Numeric Rating Scale of the pain intensity score, *SD* standard deviation

For the primary endpoint, HTX-011 subjects showed a 23% reduction in mean pain intensity over 72 h compared to saline placebo (269.39 versus 350.82; *p* < 0.0001). At all timepoints through 72 h, the mean NRS pain intensity scores were lower in the HTX-011 group when compared with saline placebo. A significant reduction of 21% for pain intensity over 72 h was observed when HTX-011 was compared to bupivacaine (269.39 versus 341.88; *p* < 0.0001). The robustness of the primary analysis was confirmed with prespecified sensitivity analysis with no adjustment of opioid use. Total opioid consumption through 72 h in the HTX-011 group was significantly reduced by 38% when compared with saline placebo (*p* < 0.0001) and by 25% when compared with bupivacaine HCl (*p* = 0.024). Overall, 51% of HTX-011 subjects were opioid-free through 72 h versus 40% for bupivacaine HCl (*p* = 0.0486) and 22% in saline placebo (*p* < 0.0001). It is notable that significantly fewer patients in the HTX-011 arm received any rescue medication through 72 h, including acetaminophen. This provides further evidence of the effectiveness of HTX-011. Beyond the 72-h timeframe, the HTX-011 group continued to show a significantly higher proportion of subjects who were opioid-free through Day 10 and through Day 28 compared with saline placebo and a numerically higher proportion of opioid-free subjects versus bupivacaine HCl. Of the 84 subjects in the HTX-011 group who were opioid-free through 72 h, 80 (95.2%) and 71 (84.5%) subjects remained opioid-free though Day 10 and Day 28, respectively.

**Fig. 2:**
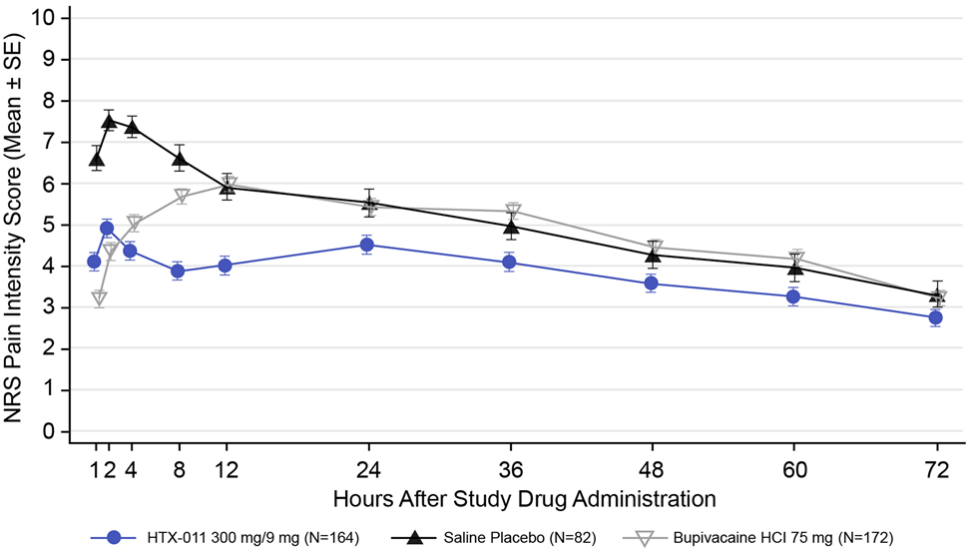
Mean (SE) NRS pain intensity scores in using wWOCF (ITT population). *HCl* hydrochloride, *ITT* intent to treat, *NRS* Numeric Rating Scale, *SE* standard error, *wWOCF* windowed worst observation carried forward

**Fig. 3:**
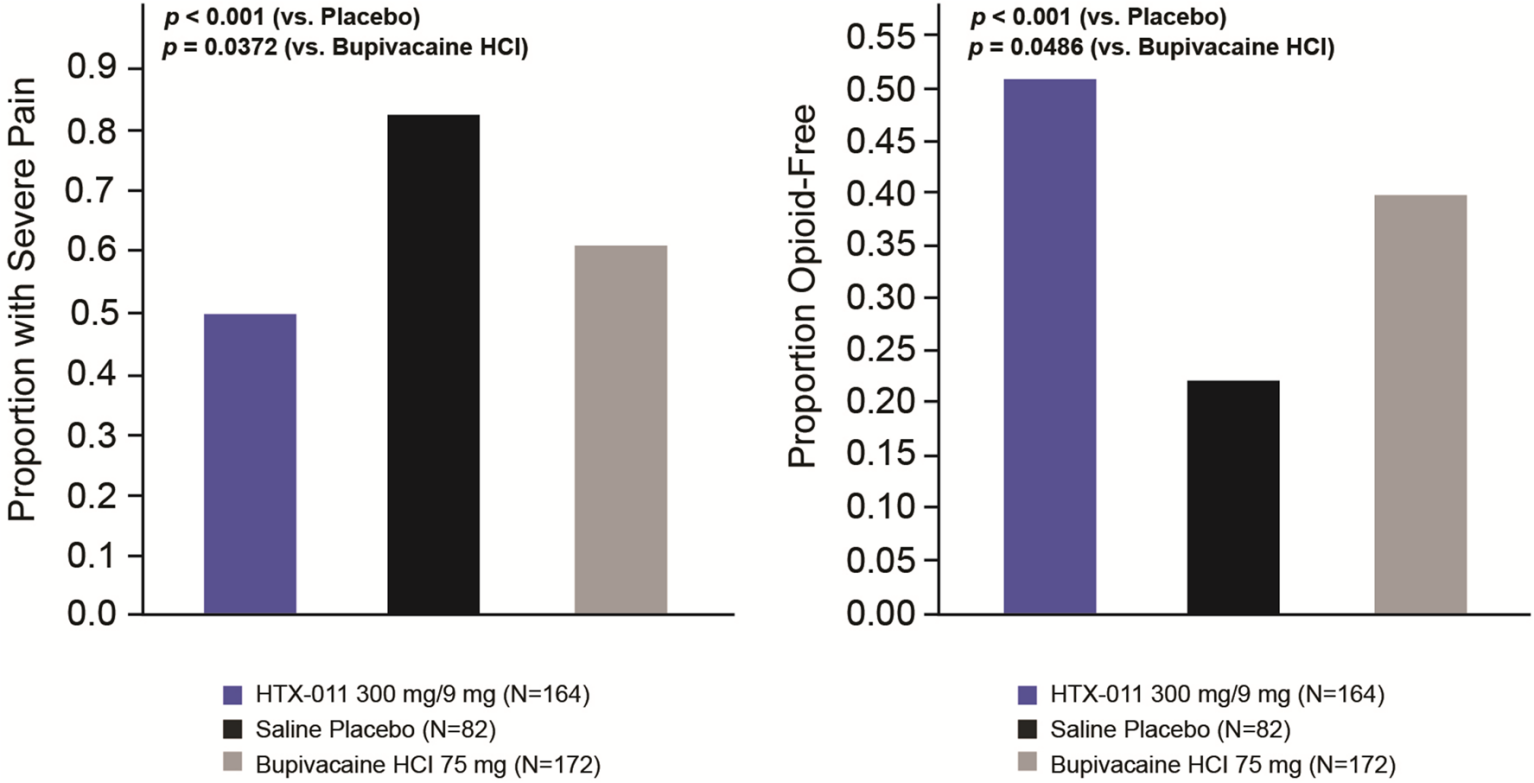
Proportion of subjects experiencing severe pain at any time from 0 to 72 h and proportion of subjects opioid-free through 72 h (ITT population). *HCl* hydrochloride, *ITT* intent to treat

A clear separation in the mean pain intensity curves throughout 72 h was demonstrated for HTX-011 compared with bupivacaine HCl and saline placebo (Fig. 2). The proportion of subjects in the HTX-011 group who experienced severe pain was significantly lower compared with bupivacaine HCl and saline placebo (Fig. 3). Specifically, fewer than half the subjects in the HTX-011 group (48.8%) experienced severe pain at any time point over 72 h compared with the 60.5% in the bupivacaine HCl group (*p* = 0.0372) and 81.7% in the saline placebo group (*p* < 0.0001).

The bupivacaine HCl group had significantly lower pain compared with saline placebo over the first 12 and 24 h; however, there was no significant difference in pain reduction between the bupivacaine HCl and saline placebo group beyond 24 h as measured by AUC_24–72_ (Fig. 2). In contrast, HTX-011 significantly reduced pain in the first 12 and 24 h versus both saline placebo and bupivacaine HCl (*p* < 0.001), and HTX-011 maintained this significant reduction beyond 24 h through the entire 72-h period (AUC_24–72_) versus both bupivacaine HCl (*p* = 0.0007) and placebo (*p* = 0.0264).

### Safety

Overall, HTX-011 was well tolerated with a safety profile comparable to placebo and bupivacaine solution (Table 4). There were no deaths and no AEs that led to study withdrawal. The four most common AEs in the HTX-011 group and in the saline placebo and bupivacaine HCl groups were nausea, constipation, dizziness, and headache (Table 4). The incidences of these AEs in the HTX-011 group were lower compared with the bupivacaine HCl group and similar or lower compared to the saline placebo group. A lower incidence of ORAEs was reported for HTX-011 (32.5%) compared with saline placebo (43.9%) and bupivacaine HCl (42.2%). There was no evidence of local anaesthetic systemic toxicity (LAST) based on a comprehensive review of potential LAST-related AEs, vital signs, ECGs, and bupivacaine plasma concentrations. Across all arms, the incidence of local inflammatory AEs was low and there was no evidence of delayed wound healing. There were also no clinically meaningful differences between HTX-011, bupivacaine HCl, and saline placebo for other safety parameters, including hematology and serum chemistry, vital signs, and ECGs.

**Table 4:** Overall summary of adverse events (safety population)

	Saline placebo (*n* = 82)	Bupivacaine HCl 75 mg (*n* = 173)	HTX-011 300 mg/9 mg (*n* = 163)
Any AE	61 (74.4%)	127 (73.4%)	119 (73.0%)
Severe AEs	2 (2.4%)	2 (1.2%)	3 (1.8%)
SAEs	1 (1.2%)	1 (0.6%)	2 (1.2%)
Deaths or fatal AEs	0	0	0
Drug-related SAEs	0	0	0
AEs leading to study withdrawal	0	0	0
Opioid-related AEs^a^	36 (43.9%)	73 (42.2%)	53 (32.5%)
Potential LAST-related AEs^b^	28 (34.1%)	71 (41.0%)	54 (33.1%)
Local inflammatory AEs^c^	2 (2.4%)	10 (5.8%)	6 (3.7%)
Most common AEs			
Nausea	28 (34.1%)	37 (21.4%)	30 (18.4%)
Constipation	15 (18.3%)	41 (23.7%)	28 (17.2%)
Dizziness	13 (15.9%)	42 (24.3%)	24 (14.7%)
Headache	10 (12.2%)	24 (13.9%)	21 (12.9%)

AEs were coded to PT using the Medical Dictionary for Regulatory Activities (MedDRA), version 19.1

Safety population includes all subjects who receive study drug. This population was used for all summaries of safety data. The actual treatment received was used for analysis in this population

*AE* adverse event, *HCl* hydrochloride, *LAST* local anaesthetic systemic toxicity, *ORAE* opioid-related adverse event, *PT* preferred term, *SAE* serious adverse event

^a^At each level of summarization (any event and PT), subjects reporting more than 1 ORAE are counted only once. ORAEs include those with sponsor-prespecified preferred terms of nausea, vomiting, constipation, pruritus, pruritus generalized, somnolence, respiratory depression, and urinary retention

^b^At each level of summarization (any event and PT), subjects reporting more than 1 potential LAST-related AE are counted only once. LAST include those with sponsor-prespecified preferred terms of any PT that includes “arrhythmia,” any PT that includes “bradycardia,” cardiac arrest, dizziness, dysgeusia, hypotension, muscle twitching, paraesthesia, paraesthesia oral, respiratory arrest, seizure, tinnitus, tremor, vision blurred, and visual impairment

^c^At each level of summarization (any event and PT), subjects reporting more than 1 potential local inflammatory AE are counted only once. Local inflammatory AEs include those with sponsor-prespecified preferred terms of blister, blood blister, cellulitis, erythema, impaired healing, incision site cellulitis, incision site complication, incision site erythema, incision site hemorrhage, incision site infection, incision site edema, incision site, rash, incision site swelling, incision site vesicles, infection, postoperative wound complications, postoperative wound infection, postprocedural cellulitis, purulent discharge, wound complication, wound dehiscence, wound infection, and wound secretion

## Discussion

HTX-011 is the first and only local anesthetic to demonstrate superior pain reduction compared to bupivacaine HCl through the critical 72-h postoperative period in a phase 3 study. The superior pain reduction was observed early in the first 24 h and importantly was sustained through 72 h. Significant reductions in overall pain and specifically in severe pain led to a significant reduction in the use of opioid rescue medication and a significant increase in the proportion of patients who remained opioid-free over 72 h. Bupivacaine is used as a local anesthetic in approximately 70% of procedures that require postoperative pain management in the United States (data on file). Therefore, we included bupivacaine HCl solution administered by standard infiltration technique as an active comparator and powered this study to demonstrate head-to-head superiority of HTX-011 over bupivacaine using a statistically rigorous approach. In the study, bupivacaine HCl 75 mg significantly reduced pain in the first 12 and 24 h versus placebo, confirming that an appropriate dose was selected as an active control. It is worth noting that based on PK modeling approximately 60 mg of bupivacaine was released from HTX-011 during the first 12 h, and this showed superior pain reduction compared to the 75-mg dose of bupivacaine HCl in the same timeframe, confirming the synergy between bupivacaine and meloxicam in HTX-011. As would be expected, there was no significant difference beyond 24 h between bupivacaine and placebo as measured using AUC_24–72_ for pain. In contrast, HTX-011 significantly reduced pain in the first 12 and 24 h versus bupivacaine and placebo (*p* < 0.001) and maintained this significant reduction versus both groups through 72 h with a significantly decreased AUC_24–72_ for pain. Most notably, a prespecified sensitivity analysis without adjustment of opioid analgesic effect confirmed the robustness of the primary analysis. Therefore, even as the control groups benefited from the pain-reducing effects of significantly greater opioid use, subjects who received HTX-011 (who utilized less opioids as well as less acetaminophen) still experienced significantly less pain through 72 h. To our knowledge, no other extended release local anesthetic, including liposomal bupivacaine, has shown significant analgesic activity in the second or third day after a single administration with or without adjustment for opioid use [6–8].

Fewer than half the subjects who received HTX-011 (49%) experienced severe pain compared with approximately 82% for saline placebo and 61% for bupivacaine HCl. This decrease in severe pain with HTX-011 was also consistent with a significant increase in the proportion of HTX-011-treated subjects who did not require opioid rescue medication over 72 h when compared with saline placebo and bupivacaine HCl (51.2% versus 22.0% and 40.1%, respectively). Furthermore, among the HTX-011-treated subjects who were opioid-free through 72 h, almost all remained opioid-free through Day 10 and this continued through Day 28. These results are impactful when interpreted in the context of contrasting findings by Wunsch and colleagues suggesting that 85.8% of opioid-naive subjects who underwent inguinal hernia repair filled a prescription for opioids within 7 days of the surgery [22]. The significant decrease in severe pain observed with HTX-011 may also have longer-term consequences, as severe postoperative pain is a known risk factor for the development of chronic inguinal pain, which can be a debilitating long-term surgical complication [1]. Further studies should be conducted to determine whether the ability of HTX-011 to reduce the proportion of subjects who experience early-severe postoperative pain leads to a reduction in chronic pain.

HTX-011 was well tolerated, with no drug-related serious AEs and fewer overall ORAEs, consistent with the prior completed phase 2 study [21]. The lower incidence of ORAEs among HTX-011-treated subjects is consistent with the lower opioid consumption and higher proportion of subjects who were opioid-free in the HTX-011 group. No cases of LAST were observed in this study and the proportion of HTX-011-treated subjects who had a potential LAST-related AE was like that in saline placebo (subjects with no exposure to bupivacaine). Moreover, the application of HTX-011 without a needle into the surgical site avoids the potential for unintended intravascular administration.

This study had some limitations. Since the typical dose for bupivacaine in herniorrhaphy can vary, the dose selected was within bupivacaine labeling expected to produce significant reductions in pain intensity. Statistically significant reductions in pain through 24 h compared with placebo were indeed observed for bupivacaine. Although pain control has been shown to be enhanced in a multimodal setting, this study was conducted to meet regulatory requirements assessing the analgesic effects of HTX-011 without scheduled postoperative analgesics. Incorporating HTX-011 into a scheduled multimodal regimen would be expected to further improve pain control and reduce opioid utilization. Lastly, to accurately collect pain data for the endpoint, this study used a 72-h inpatient period, even though patients who have undergone an open herniorrhaphy are usually discharged within a few hours. As such, it was not possible to show a significant decrease in opioid discharge prescriptions with HTX-011. Further studies are needed to confirm this important endpoint.

In summary, the first 72 h after herniorrhaphy, when pain is most severe [5], is the most crucial time to address the patient’s pain management and recovery. Effective pain management, reduced exposure to opioids, and the ability to reduce severe pain immediately following surgery have been associated with improved patient outcomes, reducing the risk for the development of chronic postoperative inguinal pain and consequent persistent opioid use [1, 3, 16]. The current study focused on patients undergoing herniorrhaphy; however, this is a well-established soft tissue pain model and, therefore, the results can be generally applicable to other soft tissue surgical procedures. HTX-011 demonstrated superior, sustained pain reduction over 72 h and significantly reduced opioid consumption in subjects undergoing herniorrhaphy compared with bupivacaine HCl and saline placebo. Furthermore, the clinical benefits of HTX-011 treatment included significantly lower proportions of subjects who experienced severe pain, significantly higher proportions of subjects who were opioid-free, and a lower proportion of subjects who experienced ORAEs. HTX-011 was well tolerated with an overall safety profile similar to saline placebo and bupivacaine. In this large, rigorously designed trial, HTX-011 demonstrated a statistical and clinically meaningful impact on both postoperative pain control and opioid consumption following inguinal herniorrhaphy.
